# Overcoming adaptive resistance in mucoepidermoid carcinoma through inhibition of the IKK-β/IκBα/NFκB axis

**DOI:** 10.18632/oncotarget.12195

**Published:** 2016-09-22

**Authors:** Vivian P. Wagner, Marco A.T. Martins, Manoela D. Martins, Kristy A. Warner, Liana P. Webber, Cristiane H. Squarize, Jacques E. Nör, Rogerio M. Castilho

**Affiliations:** ^1^ Laboratory of Epithelial Biology, Department of Periodontics and Oral Medicine, University of Michigan School of Dentistry, Ann Arbor, MI, USA; ^2^ Experimental Pathology Unit, Clinics Hospital of Porto Alegre, Federal University of Rio Grande do Sul, Porto Alegre, RS, Brazil; ^3^ Department of Oral Pathology, School of Dentistry, Federal University of Rio Grande do Sul, Porto Alegre, RS, Brazil; ^4^ Department of Restorative Sciences, University of Michigan School of Dentistry, Ann Arbor, MI, USA; ^5^ Comprehensive Cancer Center, University of Michigan Ann Arbor, MI, USA; ^6^ Department of Otolaryngology, Medical School, University of Michigan, Ann Arbor, MI, USA; ^7^ Department of Biomedical Engineering, University of Michigan College of Engineering, Ann Arbor, MI, USA

**Keywords:** salivary cancer, irradiation, IKKα, cancer stem cells, radio-adaptive

## Abstract

Patients with mucoepidermoid carcinoma (MEC) experience low survival rates and high morbidity following treatment, yet the intrinsic resistance of MEC cells to ionizing radiation (IR) and the mechanisms underlying acquired resistance remain unexplored. Herein, we demonstrated that low doses of IR intrinsically activated NFκB in resistant MEC cell lines. Moreover, resistance was significantly enhanced in IR-sensitive cell lines when NFκB pathway was stimulated. Pharmacological inhibition of the IKK-β/IκBα/NFκB axis, using a single dose of FDA-approved Emetine, led to a striking sensitization of MEC cells to IR and a reduction in cancer stem cells. We achieved a major step towards better understanding the basic mechanisms involved in IR-adaptive resistance in MEC cell lines and how to efficiently overcome this critical problem.

## INTRODUCTION

Salivary gland cancer (SGC) is a relatively rare group of tumors, with annual incidence rates between 0.05 to 2 new cases per 100,000 [1]. In the US, the incidence of SGC significantly increased from 1974–1976 to 1998–1999 and accounted for 6.3% compared to 8.1% of all head and neck cancers, respectively [2]. The 5-year survival rate for SGC is 60–80%; however, this rate drops to 50% by 10 years [3]. Distant metastasis, more frequently in the lungs, is the primary cause of death and occurs slowly, with patients surviving up to 20 years [1]. Mucoepidermoid carcinoma (MEC) is the most common malignant SGC, followed by adenoid cystic carcinoma [4–6]. Treatment for MEC derives from therapeutic protocols optimized for head and neck squamous cell carcinomas [7]. Surgical excision and ionizing radiation (IR) are the first-line treatment options for resectable and unresectable tumors, respectively. Postoperative IR is recommended for patients with residual disease with extensive nodal metastasis or capsular rupture. Postoperative radiotherapy is also suitable for patients with high-grade tumors and advanced disease, positive margins and perineural or vascular invasion [1]. In general, more than 80% of all SGC patients receive radiotherapy [8]. Although IR therapy used broadly to treat SGC, including MEC, little is known about how resistance to radiation develops in SGC cells. Moreover, the low survival rates that occur in the long-term underscore the urgent need to identify molecular targets that sensitize SGC cells to radiotherapy.

Nearly 50% of all cancer patients will be treated with IR alone or in combination with surgery or chemotherapy [8]. IR activates the DNA damage response pathway and cell cycle arrest, leading to senescence or apoptosis [9]. The responsiveness of the tumor to IR is substantially mediated by the intrinsic radiosensitivity of tumor cells [10]. Fractionated radiotherapy enables normal tissues to recover, but it also allows the surviving fraction of tumor cells to proliferate, promoting long-term resistance [11]. Distinct pathways, such as the NFκB pathway, are triggered during radiotherapy. Activation NFκB leads to increased cellular tolerance to subsequent IR doses in various cell lineages, such as breast, prostate and lung cancer cells [12, 13]. The NFκB canonical or classical, pathway is activated by a pro-inflammatory stimulus, such as TNF-α, that triggers the activation of the IKK complex. Activated IKK-α and IKK-β phosphorylate IκB-α at S-32 and S-36, allowing NFκB to translocate to the nucleus where it acts as a nuclear transcription factor [14, 15]. Cancer cells normally have high NFκB activity, [16] leading to increased cell survival via antagonism of apoptotic pathways [17]. Indeed, the NFκB subunit RelA (p65) can promote resistance to programmed cell death by suppressing p53 function [18]. We have also show that NFκB signaling drives chemoresistance in head and neck squamous cell carcinoma by modulating chromatin modifications [19].

Previous studies showed that both low and high doses of IR upregulate NFκB binding activity in several types of solid tumors in a dose- and time-dependent manner [13, 20, 21]. The level of NFκB in tumor cells is an important determinant of responsiveness to IR because NFκB induces resistance in several tumor models by inhibiting apoptosis after DNA damage [13, 22]. Activated NFκB regulates the transcription of over 400 genes, including Bcl-2, BcL-xL, XIAP, survivin and AKT, which are associated with NFkB-driven radioresistance [23]. Recently, it was shown that IKK-β regulates the repair of DNA double-strand breaks induced by IR in breast cancer cells [15]. This evidence supports the use of NFκB inhibitors as adjuvant treatment to sensitize cancer cells to IR. In fact, promising pre-clinical results have been achieved in colorectal cancer [24], melanoma [25] and neuroblastoma [26]. Current therapeutic strategies focused on inhibiting NFκB signaling rely on proteasome inhibition, resulting in off-target effects. Identifying new drugs that induce selective inhibition of NFκB, by interfering with IKKs or by inhibiting phosphorylation and promoting loss of function of the IκB-alpha super-repressor, are expected to efficiently reduce tumor resistance.

In our study, we explored the response of MEC cells to IR. We found that high intrinsic radioresistance of MEC cells is associated with NFκB activation. Furthermore, inhibition of NFκB using FDA-approved Emetine resulted in targeted inhibition the NFκB super-repressor IκB alpha. Emetine also disrupted cancer stem cells (CSC) by inhibiting the Ikk-β subunit and inducing apoptosis.

## RESULTS

### Ionizing radiation differentially affects mucoepidermoid carcinoma cell lines

**Figure 1:**
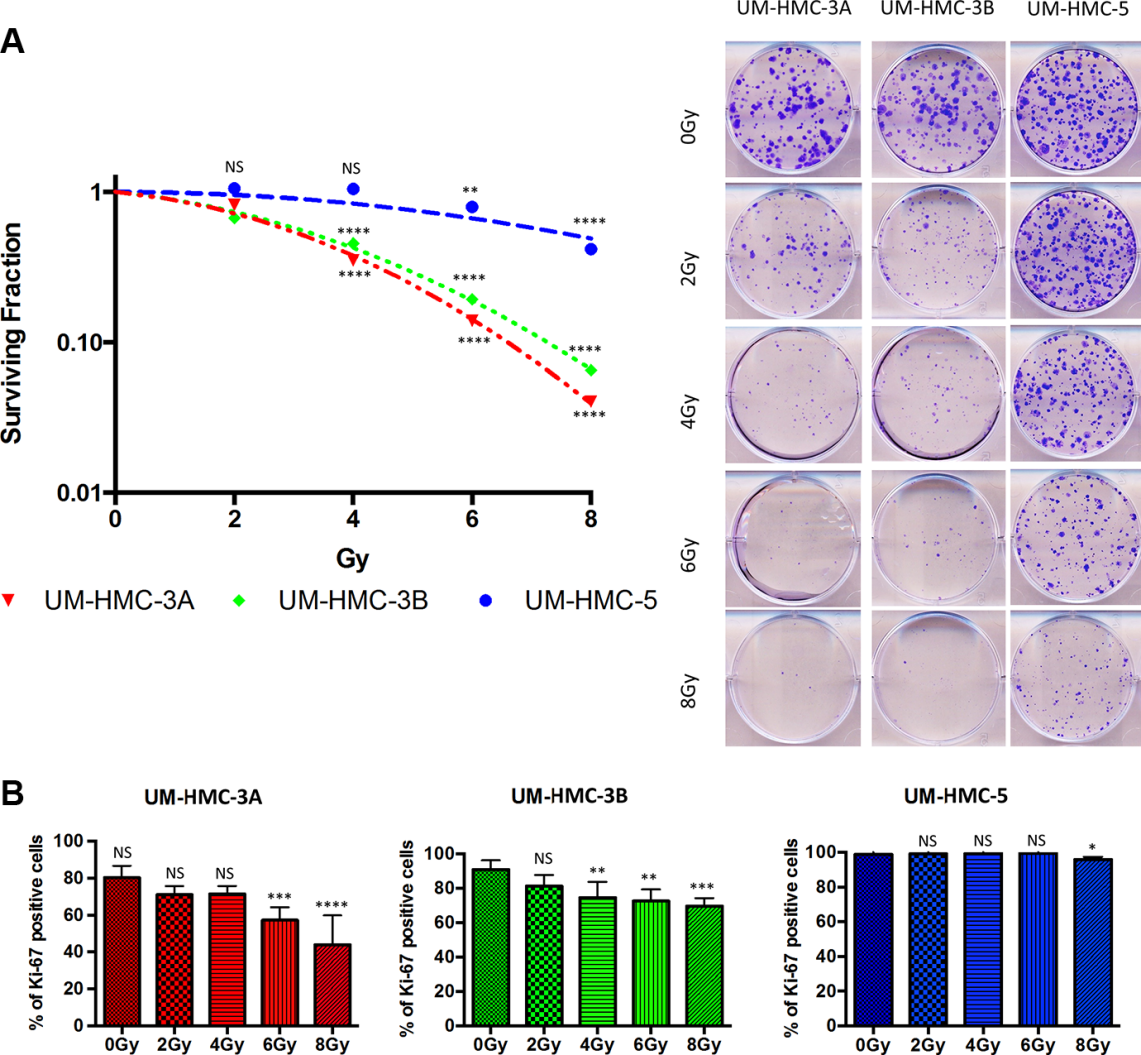
Intrinsic resistance of MEC cells to IR (**A**) Clonogenic survival was measured 7 days after IR (0–8 Gy). The data are expressed as mean ± SD (*n* = 3) of survival fraction compared to nonirradiated cells. Colonies consisting of more than 50 cells were scored as surviving colonies. (**B**) Cells were stained for Ki67 24 hrs after IR, scored for positive nuclear staining and presented as a percentage of the total cell number (*n* = 3).

SGC are historically recognized to be radioresistant. Nevertheless, IR is widely used for the treatment of advanced and high-grade tumors. This historical concept is based on cross-sectional studies of lung cancer that observed poor local control rates of tumors receiving conventional doses of radiotherapy [27]. Little is known about the radiosensitivity of SGC given the small number of available tumor cell lines. For the first time, we evaluated the response of three different MEC cell lines, recently established at the University of Michigan School of Dentistry, [28, 29] using a wide range of IR doses (0, 2, 4, 6 and 8 Gy). Radiosensitivity differed among the cell lines (Figure 1A). The most sensitive MEC cell lines (UM-HMC-3A and UM-HMC-3B) had a survival fraction at 2 Gy (SF_2_) of 0.81 while UM-HMC-5 had an SF_2_ of 0.97. Previous studies have shown that other carcinomas cells, such as cervical squamous cell carcinoma, have a much lower SF_2_ (0.27 to 0.75) compared to our MEC cell lines [30]. Despite UM-HMC-3A and UM-HMC-3B cells having similar SF_2_ values, the metastatic lymph node UM-HMC-3B cells had increased resistance to IR at intermediate levels of radiation (4 and 6 Gy, ***p < 0.001) (Figure 1A).

To further characterize the resistance phenotype observed in MEC cells, we examined their proliferation in response to various doses of radiation. To our surprise, UM-HMC5 radioresistance phenotype was directly associated with the maintenance of basal levels of cellular proliferation, as measured by Ki67 (Figure 1B). Reduced proliferation was only observed in response to high doses of IR (8 Gy, *p < 0.05). We observed a statistically significant reduction in proliferation at smaller doses of IR (4 Gy and 6 Gy) in UM-HMC-3A and UM-HMC-3B cells, respectively. Interestingly, a low dose of IR (2 Gy) induced an increase in the number of abnormal mitotic figures in UM-HMC-5 cells that is defined by the presence of multipolar, ring, dispersed, asymmetrical and lag-type mitoses (Supplementary Figure S1).

### Mucoepidermoid carcinomas express high basal levels of NFκB

**Figure 2:**
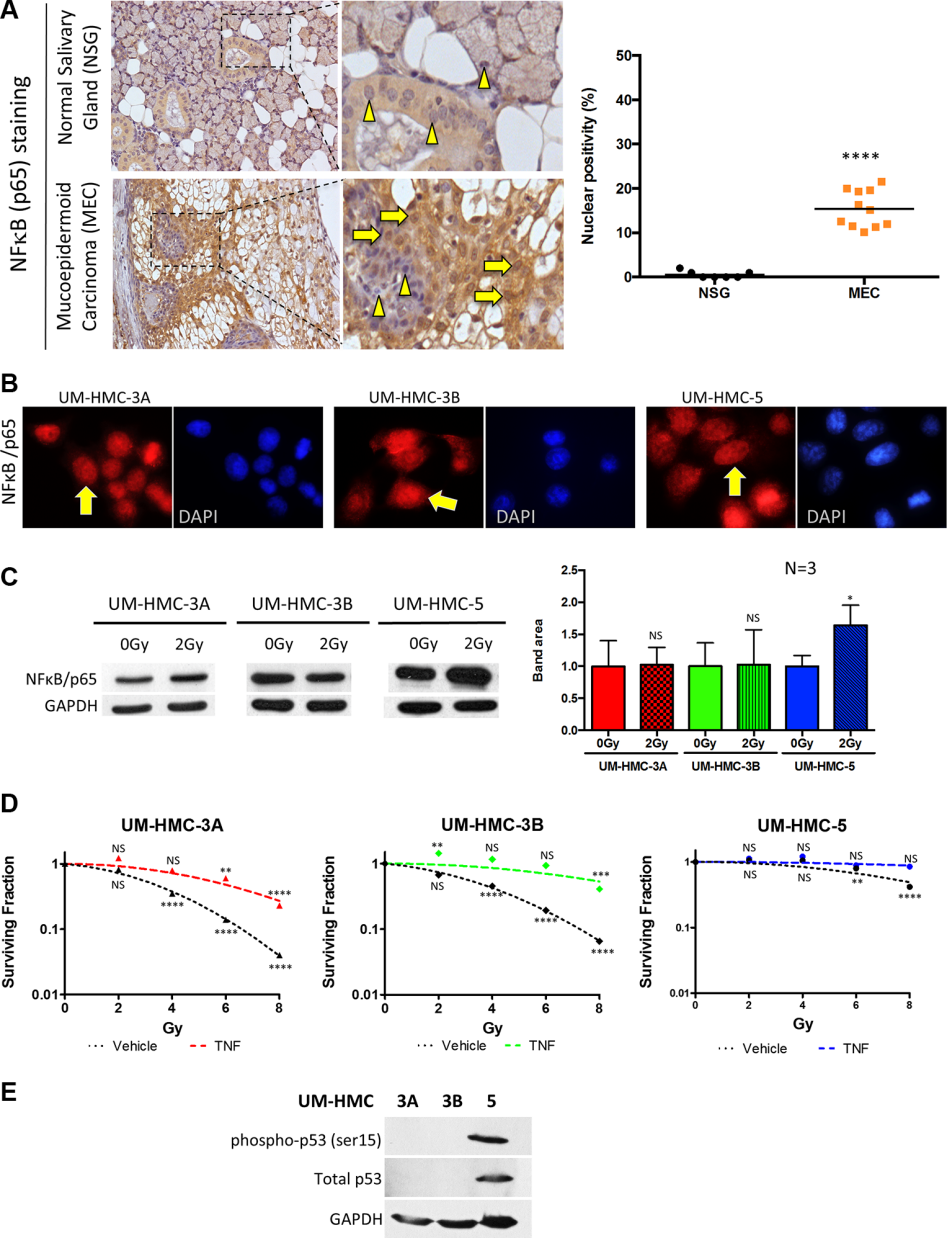
Activation of NFκB in MEC (**A**) NFκB (p65) (yellow arrows) was significantly increased in the nucleus of MEC samples compared to normal salivary glands, which showed prevalent cytoplasmic staining (arrowhead) (***p < 0.001, *n* = 11, mean ± SD). (**B**) Immunofluorescence of UM-HMC-3A, UM-HMC-3B, and UM-HMC-5 tumor cell lines depict the presence of nuclear NFκB (yellow arrows). (**C**) UM-HMC-3A, UM-HMC-3B, and UM-HMC-5 show detectable NFκB (p65) protein levels at baseline (0 Gy). NFκB is increased in UM-HMC-5 following 2 Gy of IR (*p < 0.05, mean ± SD from experiments run in triplicate). (**D**) Clonogenic assay for MEC cells with no stimuli or TNF-α stimuli revealed that NFκB upregulation significantly increases the resistance of UM-HMC-3A and UM-HMC-3B (**p < 0.01, ***p < 0.001, *n* = 3, mean ± SD compared to 0 Gy). (**E**) Western blot of UM-HMC-3A, UM-HMC-3B, and UM-HMC-5 for phosphorylated p53 (ser15) depict high expression of the p53 protein on UM-HMC-5 cells. UM-HMC-3A and 3B are absent of p53 protein levels.

NFκB is a crucial player in several steps of cancer initiation and progression, primarily due to its strong anti-apoptotic effect in cancer cells [31]. In most cell types, NFκB dimers are predominantly inactive in the cytoplasm; however, cancer cells typically have high NFκB activity [16]. We analyzed the protein expression of nuclear NFκB (active form) in human samples of MEC and normal salivary gland (NSG). Interestingly, although NFκB is predominantly cytoplasmic in NSG samples (Figure 2A arrowhead; mean 0.5% of nuclear staining/sample), all MEC samples were positive for nuclear NFκB (10.1% – 20.5% of nuclear staining/sample) (Figure 2A arrow). Further, we have explored the presence and localization of NFκB in our MEC cell lines. Similar to the observed in paraffin sections of human MEC tumors, all cell lines expressed nuclear NFκB (Figure 2B). Nuclear NFκB is associated with poor prognosis in several cancers, including rectal [32], esophageal [33] and head and neck cancers [34]. In adenoid cystic carcinomas, NFκB expression is considered an independent prognostic factor associated with poor overall survival [35]. Although no clinical association could be established in our samples, the increase in active NFκB suggests that this pathway plays a role in MEC behavior. However, it is unknown whether high basal levels of NFκB are associated with resistance to radiotherapy in MEC.

### IR induces accumulation of NFκB and activation of the NFκB signaling pathway induces IR resistance

To better understand the correlation between NFκB expression and tumor resistance to radiotherapy, we examined the effects of IR on NFκB activity in MEC. We exposed our three MEC cell lines to 2 Gy of radiation, which is the daily faction dose recommended for MEC patients receiving radiotherapy [1]. As revealed by Western Blotting, all MEC cell lines had detectable levels of NFκB at baseline (0 Gy), corroborating to our findings from patient samples that showed detectable levels of NFkB. Interestingly, 2 Gy IR-induced the accumulation of NFκB in UM-HMC-5, but had no effect on NFκB in UM-HMC-3A and UM-HMC-3B (Figure 2C, *p < 0.05), corroborating to our previous finding that UM-HMC-5 have a higher resistance profile. Notably, our findings suggest that certain MEC patients may not benefit from fractionated radiotherapy; in contrast, 2 Gy IR may stimulate radio-adaptive resistance through NFκB signaling. In addition, administration of the clinically relevant 2 Gy dose resulted in a substantial increase in mitosis, including the presence of aberrant mitotic figures (Supplementary Figure S1, arrows **p < 0.01).

We next explored whether upregulation of NFκB directly influences MEC resistance to IR. Active NFκB signaling induces anti-apoptotic proteins, resulting in tumor cells evading radiotherapy [12]. Using a clonogenic assay, we found that stimulation of the NFκB pathway using TNF-α led to increased resistance of UM-HMC-3A and UM-HMC-3B tumor cells to IR (Figure 2D). Interestingly, UM-HMC-5, which previously showed elevated radioresistance (Figure 1A) and NFκB levels (Figure 2C NS *p* > 0.05, *p < 0.05) in response to IR, did not benefit from TNF-α (Figure 2D). We observed that NFκB activation markedly increased the resistance of MEC cell lines to IR. In response to all IR doses, UM-HMC-3A and UM-HMC-3B cells stimulated with TNF-α were more resistant than the control (Figure 2D, NS *p* > 0.05, **p < 0.01, ***p < 0.001). Our findings suggest that UM-HMC-5 cell respond to low doses of radiation by inducing the activation of the NFκB signaling pathway, and that administration of TNF-α does little to further activate the NFκB signaling on UM-HMC-5 when compared to the UM-HMC-3A and 3B cell lines. Although we have established a correlation between the NFκB pathway and MEC resistance to radiotherapy, the clinical relevance of inhibiting this pathway in MEC is unknown.

In search for a potential mechanism associated with increased resistance to chemotherapy of UM-HMC-5 cell line, we explored the p53 status of our MEC cell lines. We used a phospho-p53 antibody phosphorylated at the serine 15 that is associated with p53 gain of function (active). Indeed, we found that p53 is highly expressed in UM-HMC-5 cell line compared to UM-HMC-3A and 3B cells in which the p53 levels could not be detected (Figure 2E). Similar to the accumulation of phosphorylated p53 protein, the total amount of p53 protein (not phosphorylated) was also very high compared to UM-HMC-3A and 3B. It has been demonstrated that p53 gain of function is often associated with mutations and constitutive expression of mutant p53 interferes with the process of apoptosis, a major and essential event for the success of any anticancer treatment. While p53 wild type cell lines are more sensitive to DNA damaging agents, mutant p53 confers resistance to DNA-damage related apoptosis [36]. Besides, p53 protein is responsible for prolonged arrest following IR exposure, thereby facilitating the DNA repair in the absence of apoptosis [37]. The absence of p53 observed in UM-HMC-3A and 3B cell lines support the notion that tumors presenting inactivate p53 lack the ability to repair the DNA. Lack of p53, and increased sensitive to DNA-damage result in mitotic catastrophe, the main antitumor mechanism associated to irradiation [37].

### Emetine-induced inhibition of NFκB is mediated by downregulation of IκB-α/IKK-β and p21

**Figure 3:**
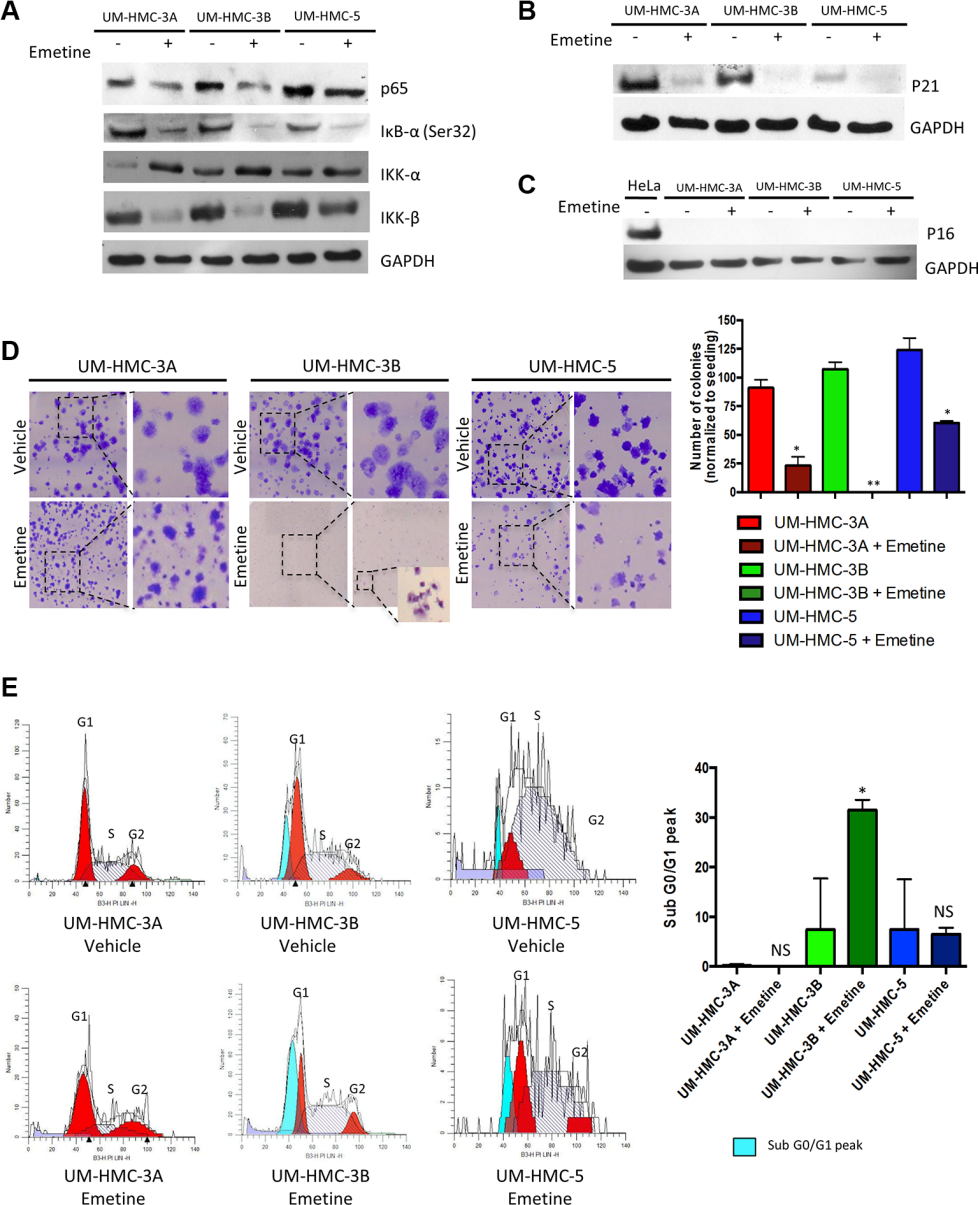
Emetine induces inhibition of the IKK-β/IκB-α/NFκB axis and induces apoptosis of UM-HMC-3B (**A**) Down-regulation of NFκB expression in MEC cells after Emetine treatment for 24 hrs was confirmed by Western blot analysis. Emetine inhibited IκB-α (Ser32) and IKK-β phosphorylation but did not affect the IKK-α subunit. (**B**) Emetine treatment for 24 hrs lead to p21 inhibition, and (**C**) had no effect on p16. (**D**) Emetine disrupted the colony forming potential of MEC cells. UM-HMC-3A and UM-HMC-5 treated for 24 hrs with Emetine had a significantly smaller number of colonies after 7 days in culture while UM-HMC-3B did not have colonies larger than 50 cells. (**E**) Emetine causes cell cycle arrest at the sub G0/G1 checkpoint in UM-HMC-3B cells (*p < 0.05).

Targeted inhibition of NFκB is a promising novel adjuvant treatment for sensitizing cancer cells to radiotherapy. Encouraging results have been reported in different solid tumors, such as colorectal [24] and prostate [38] cancer. However, FDA-approved NFκB inhibitors, such as Bortezomib, are proteasome inhibitors that target the multi-catalytic proteinase complex involved in protein degradation. Inhibitors like Bortezomib downregulate NFκB but also increase targeted activity of cell cycle proteins and apoptosis-associated pathways [39]. Recently, Emetine, a drug purified from the ipecac root, was shown to be a novel selective inhibitor of NFκB [40]. FDA-approved Emetine has emetic properties and has been used for decades to treat protozoan infections. Emetine selectivity inhibits IκBα phosphorylation at Ser32, thereby preventing NFκB from translocating to the nucleus and altering gene expression [40]. We assessed the ability of Emetine to inhibit the NFκB pathway in MEC tumor cells. We found that Emetine efficiently reduced phosphorylated IκB-α and NFκB protein (Figure 3A). Surprisingly, Emetine downregulated IKK-β, but not IKK-α, subunit protein (Figure 3A). IKK-β activation triggers the canonical NFκB pathway [41] and regulates several pro-survival and anti-apoptotic genes, including Bcl2, Bcl-XL, and XIAP [42]. Interestingly, the effect of Emetine on IKK-β has not been observed in other systems. Our results also demonstrated that Emetine downregulated p21 expression. Although earlier studies suggested that p21 suppresses cancer through promotion of cell cycle arrest, cellular differentiation, and senescence, recent studies suggest that p21 induces proliferation and cellular transformation and is associated with poor prognosis in the prostate, ovarian, cervical, breast, brain, and esophageal squamous cell carcinomas [43–50]. Indeed, MEC tumor cell lines expressing p21 (Figure 3B) failed to activate senescence (Figure 3C), as measured by p16^ink4^ levels, in basal conditions and following administration of Emetine, suggesting that p21 acts as an oncogene in MEC, as it does in other cancers. Supporting our findings, Emetine downregulated p65 and IKK-β and further suppressed p21, leading to reduced colony formation in all analyzed MEC cell lines (Figure 3D, *p < 0.05; **P < 0.01). Although we have not assessed if p53 is mutated in our cell lines, the data show an interesting pattern previously demonstrated by the group of Manuel Serrano in head and neck cancers, in which high levels of p21 do not correlate to p53 levels but does correlate with better survival rates [51], and in our case, more sensitize tumor cells to radiation (UM-HMC3A and 3B). In Ovarian cancer, the group of Berchuck has shown that the presence of mutated p53 is usually associated with decreased p21 expression [52]. Our findings suggest a potential p53 dependent mechanism associated to UM-HMC-5 resistance to radiotherapy. Notably, MEC cells are sensitive to NFκB inhibition, suggesting tumor progression is dependent on this pathway. Indeed, as compared to UM-HMC3A and UM-HMC5 cells, the metastatic UM-HMC3B cells were so sensitive to Emetine that we could not identify the formation of tumor colonies (Figure 3D). Impaired colony formation was due, in part, to the activation of apoptosis in UM-HMC3B cells, as shown by the SubG0/G1 peak during cell cycle analysis (Figure 3E).

### Inhibition of the IKK-β/IκB-α/NFκB signaling axis sensitizes MEC cells to IR

**Figure 4:**
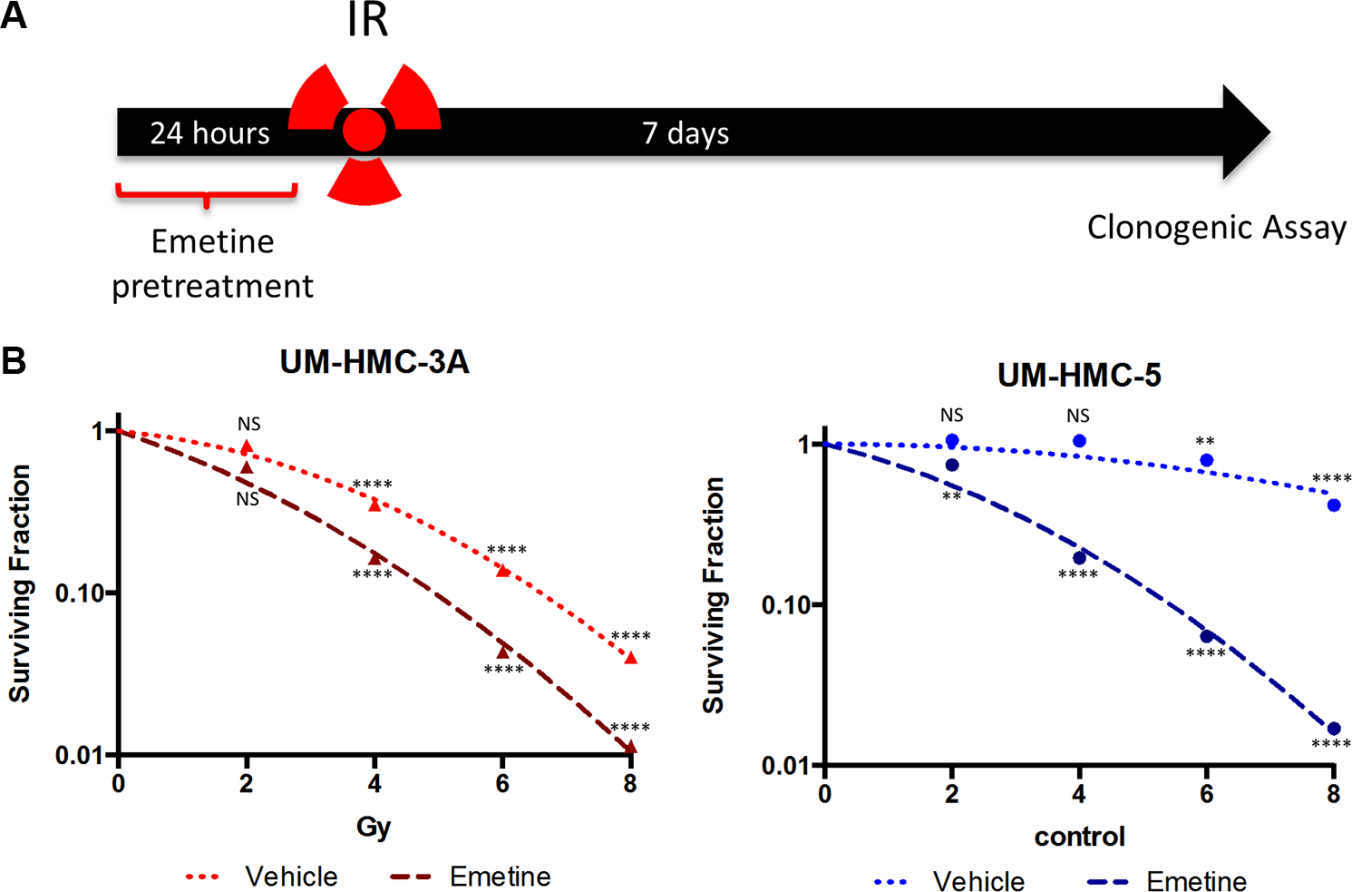
Pharmacological inhibition of NFκB sensitizes MEC cells to IR (**A**) Cells were sensitized with Emetine for 24 hrs before IR. The drug was removed before IR exposure, and the clonogenic assay was performed after 7 days. (**B**) Survival was significantly decreased in UM-HMC-3A and UM-HMC-5 sensitized with Emetine compared to vehicle (*n* = 3, mean ± S, D compared to 0 Gy).

Following our previous finding that activation of the NFκB pathway increases MEC resistance to IR, we hypothesized that inactivation of the pathway by Emetine would sensitize cells to IR. To test this hypothesis, we treated all cell lines with Emetine 24 hours before irradiation. To properly understand the therapeutic efficacy of Emetine as a sensitizing agent, we removed Emetine from the culture media prior to irradiation (Figure 4A). Tumor cells were allowed to grow for 7 days before we assessed colony formation. Control tumor cells received radiation alone. We observed major declines in the surviving fraction at all IR doses when NFκB was inhibited prior to irradiation (Figure 4B). When UM-HMC-3A and UM-HMC-5 were sensitized with Emetine, we achieved a mean improvement in the SF_2_ of 24.8%. UM-HMC5 cells, originally very resistant, were sensitized to irradiation following NFκB inhibition (Figure 4B). Collectively, our data suggests that NFκB activation promotes IR resistance, and that pharmacological inhibition of NFκB sensitizes MEC cells to IR.

### Emetine-induced inhibition of the IKK-β/IκB-α/NFκB signaling axis potentialize the ability of IR to deplete MEC cancer stem cells (CSCs)

**Figure 5:**
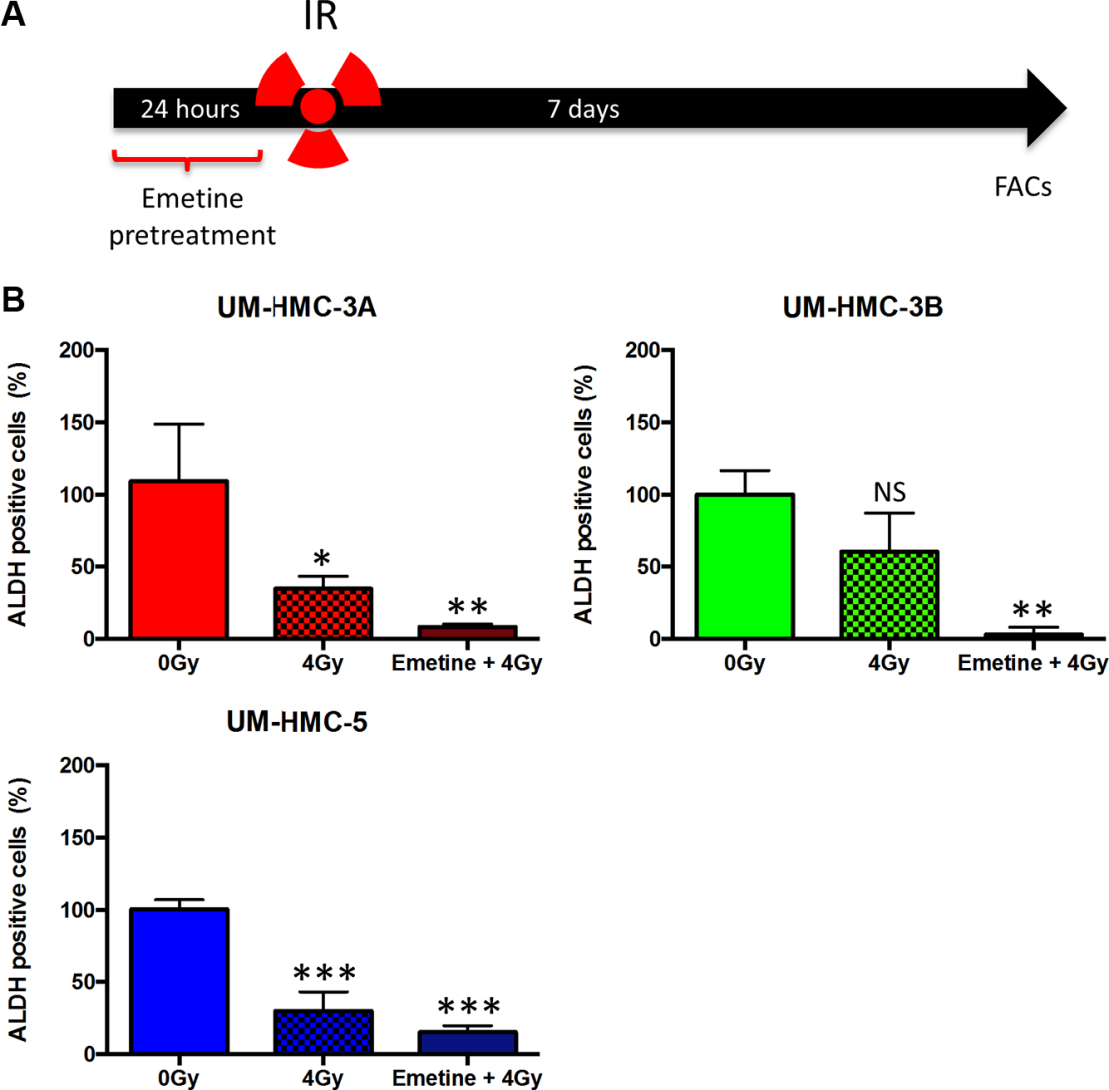
A combination of NFκB inhibition and IR efficiently deplete CSCs (**A**) Cells were sensitized with Emetine for 24 hrs; Emetine was removed from the media before IR. Cells were collected and processed for ALDH enzymatic activity using fluorescence-activated cell sorting (FACS). (**B**) A combination of NFκB inhibition and IR leads to enhanced depletion of CSCs in MEC cell lines compared to radiation alone (*n* = 3, mean ± SD).

Among the mechanisms involved in the acquisition of resistance in cancer cells are the activation of NFκB signaling and the presence of CSCs [53–55]. CSCs represent a subset of tumor cells that have stem cell-like proprieties, such as self-renewal and multipotency. Recently, Adams et al. demonstrated that MEC contains a small population of CSCs with enhanced tumorigenic potential [29]. Both NFκB and CSCs are closely interrelated and inhibition of the NFκB pathway blocks the expression of genes associated with stem cells, including Nanog and Sox2 in mammary cells [56]. Furthermore, canonical and noncanonical NFκB signaling drives CSC maintenance in breast cancer cells [57]. We found that targeted inhibition of NFκB resulted in sensitization of MEC tumor cells to IR (Figure 4B), but we did not know whether Emetine would affect CSCs. Using a similar approach described earlier, we administered Emetine 24 hours before IR (Figure 5A). Seven days after IR, tumor cells were collected and processed for ALDH enzymatic activity using fluorescence-activated cell sorting (FACS). Interestingly, combined administration of Emetine and IR resulted in further depletion of CSCs in all MEC tumor cells compared to radiation alone (Figure 5B). Radiation alone failed to reduce CSCs in UM-HMC3B cells (ns *p* > 0.05) and only slightly, but significantly, reduced CSCs in UM-HMC3A cells (*p < 0.05). However, sensitizing the cells with Emetine resulted in a significant reduction in CSCs in UM-HMC3A and UM-HMC3B MEC cells compared to controls (0 Gy) (**p < 0.01). CSCs were significantly reduced in UM-HMC5 cells in response to radiation alone compared to controls (0 Gy) (***p < 0.001); nonetheless, Emetine further reduced CSCs in UM-HMC5 cells (Figure 5B ***p < 0.001). Our findings demonstrate that inhibition of the NFκB signaling pathway in MEC tumors is an effective therapeutic strategy to sensitize tumor cells to radiation independent of the initial resistance of each cell line to radiation.

## DISCUSSION

MEC represents the most common malignant SGC [4–6]. In contrast to other glandular tumors, significant advances in treatment and overall survival for SGC patients have not improved in the last three decades. Surgery remains the first-line therapy option for MEC. Tumors located in the parotid gland usually require superficial or radical parotidectomy for infiltrative cases, compromising the maintenance of the facial nerve [1]. For minor SGC, commonly located in the palate, maxillectomy is typically required [58]. The amount of sequels and high morbidity, which is associated with low survival rates over long-term periods, underscore the need to identify therapies that improve survival and quality of life. It is estimated that more than 80% of SGC patients will need radiotherapy as first-line or adjuvant therapy [8]. However, both intrinsic resistance of MEC cells to IR and the basic mechanisms underlying acquired resistance remain unexplored. We have provided initial evidence regarding the molecular response of MEC cells to IR. We showed that radioresistance of MEC is NFkB-dependent and that targeting this pathway with Emetine improves the efficiency of IR *in vitro*. Administering a single dose of Emetine before IR sensitizes the majority of the tumor cells, including CSCs.

Emetine is a natural crystalline alkaloid found in ipecac syrup, which is derived from *Psychotria ipecacuanha.* Emetine has been widely used to treat amoebiasis since the early 1900s [59]. The earliest report describing the use of Emetine as an anti-neoplastic drug dates to 1918; however, Phase I/II clinical trials using emetine were not performed by the NCI until the mid-1970s [60–62]. Due to disparate results with Emetine, ranging from no clinical benefit to disease stabilization/tumor regression, it was not widely studied for its anti-neoplastic properties for many years. It was not until the 2000s that new reports examining the effect of Emetine on neoplastic cells emerged [63–69]. The identification of new therapeutic applications of already approved drugs is referred to as drug repurposing. Given that 80% of new drugs that enter human clinical testing are never approved for use, repurposing has the advantages of reduced safety risks, faster access to treatment and decreased cost [70, 71]. Because more than $90 billion is spent on the development of oncology drugs, and it takes more than 14 years for a promising new molecule to be translated to an approved drug, the National Center for Advancing Translational Sciences (NCATS) at NIH in funding projects that can provide Phase I/II proof-of-principle data using repurposed drugs [72]. Previous studies demonstrated that Emetine induces apoptosis in ovarian carcinoma [66], leukemic [64] and pancreatic cells [69] and arrests growth of bladder cancer cells [67]. Moreover, the daily subcutaneous dose of 1 mg/kg Emetine does not cause toxicity in patients [61], highlighting its safety in humans. A recent study by Miller et al. showed that Emetine inhibits IκB-α phosphorylation [40] but, to our knowledge, we are the first to examine this effect in cancer treatment.

Reactive oxygen species (ROS) and increased DNA damage triggered by IR activates nuclear ataxia telangiectasia mutated (ATM) signaling, resulting in activation of the IKK complex [12]. An active IKK complex induces IκB degradation and translocation of NFκB to the nucleus [16]. In the early 2000s, it was found that NFκB activation is associated with radioresistance in breast cancer cells following fractionated IR treatment [73, 74]. A recent study showed that NFκB activation protects from radiation in human Ewing sarcoma, neuroblastoma, breast, bladder, colon, prostate and lung cancer cells. In this study, high doses of IR (4 Gy) induced cell death; nevertheless, a previous study showed that sub-lethal doses of IR (2, 10, 50 or 100 cGy) induced NFκB activation and prevented cell death compared to higher doses of IR [13]. We demonstrated that a low-dose of IR (2 Gy) activated NFκB in the most resistant MEC cell line. Further, when the NFκB pathway was activated in MEC cells through a TNF-α stimulus, resistance was enhanced in the most IR-sensitive cell lines. These findings significantly advance our understanding of IR-adaptive resistance in MEC cells, a field not previously explored. Also, our findings suggest that radioresistant MEC tumors are likely to respond to radiation by overexpressing NFκB signaling to levels that confer radioresistance, similar to the achieved upon administration of TNF-α (Figure 2D). Inhibition of NFκB signaling in combination with IR may be a novel treatment for MEC. We showed that disrupting the NFκB pathway using Emetine before IR significantly increased the sensitivity of MECs to IR and that Emetine combined with IR decreased CSCs.

CSCs are a subset of tumor cells that self-renew and are multipotent with the ability to generate heterogeneous lineages of neoplastic cells that comprise the tumor. Thus, treatment will only be successful if it destroys CSCs, and this needs to be considered when optimizing anti-neoplastic drugs so as not to underestimate curative potential. Numerous findings suggest that intrinsic tumor radioresistance is associated with a higher proportion of CSCs [75–77]. Kurth et al. found that ALDH+ head and neck cancer cells maintain their tumorigenic properties after irradiation, increasing the chances of recurrence [54]. Failure to eradicate CSCs after IR may result in tumor recurrence and neoplastic cell dissemination, leading to local or distant metastasis. IR alone had no effect on CSCs in our metastatic cell line (UM-HMC-3B), which is alarming given that one of the main indications for IR treatment in MEC patients is the presence of advanced disease [1]. Targeting NFκB to disrupt CSCs is based on evidence that inhibition of NFκB downregulates genes associated with stemness proprieties, such as Nanog and Sox2 [56]. By sensitizing MEC cells to IR using Emetine, we were able to eradicate CSCs. Our findings strongly support the combination of NFκB inhibition and IR as a promising treatment option for MEC patients because it targets the bulk of the tumor in addition to CSCs.

To establish new strategies that improve the efficacy of IR, we must understand the biological factors involved in radiotherapy outcomes. While various pathways have been associated with radioresistance in different types of tumors, we must consider tumor specificity. Certain tumor types may benefit from a specifically targeted inhibition that is not successful in other types of tumors. Until now, the mechanisms underlying MEC radioresistance were unknown. We showed that sensitizing MEC cells with Emetine improved the SF_2_, the most relevant IR dose in the clinic, by 24.8%. Tumor control rates can be improved by 5–30% by increasing the effective dose IR by just 10% (reviewed in [22]). Our study highlights the importance of Emetine as a sensitizer agent to radiation. Although these results bring an encouraging and promising therapeutic strategy to manage MEC patients, it's important to emphasize that *in vivo* studies are necessary to confirm our preliminary data.

## MATERIALS AND METHODS

### Human tissue specimens

Cases of MEC diagnosed between January 1995 and December 2010 were retrieved from archives of the Pathology Service of Clinic Hospital in Porto Alegre, Rio Grande do Sul, Brazil (Human Research Ethics Committee approval: 11739012.1.0000.5327). The original hematoxylin-eosin stained slides were reviewed to confirm the diagnosis.

### Immunohistochemistry

MEC samples were sectioned into 3-μm sections, deparaffinized in xylene and hydrated in descending grades of ethanol. Endogenous peroxidase activity was blocked using 5% hydrogen peroxide in two 15-minute baths. The avidin-biotin blocking kit was used to block nonspecific binding (Kit Vector Laboratories, Burlingame, CA, USA). Slides were incubated overnight with anti- NFκB p65 (BD Biosciences, Mountain View, CA, USA) and then incubated with diaminobenzidine tetrahydrochloride (DAB, Sigma-Aldrich, St. Louis, MO, USA) and counterstained with Mayer's hematoxylin.

### Cell lines

MEC cell lines UM-HMC-3A, UM-HMC-3B and UM-HMC-5, were initially described by Warner et al. (2013). Cells were maintained in a 5% CO_2_ humidified incubator at 37°C and cultured in RPMI 1640 (Thermo Scientific, Waltham, MA, USA) supplement with 10% Fetal Bovine Serum (Thermo Scientific), 1% antibiotic (Invitrogen, Carlsbad, CA, USA), 1% L-glutamine (Invitrogen), 20 ng/ml epidermal growth factor (Sigma–Aldrich), 400 ng/ml hydrocortisone (Sigma–Aldrich) and 5 μg/ml insulin (Sigma–Aldrich). UM-HMC-3A, UM-HMC-3B, and UM-HMC-5 were treated with 0.10 μM, 0.26 μM, and 0.08 μM of Emetine dihydrochloride hydrate (Sigma–Aldrich), respectively, and 10 ng/ml of TNF-α (PeproTech, Rocky Hill, NJ, USA).

### Ionizing radiation (IR)

Ionizing radiation (IR) was performed at a dose of approximately 2 Gy/min using a Philips RT250 (Kimtron Medical, Oxford, CT, USA) in the University of Michigan Comprehensive Cancer Center Experimental Irradiation Core (Ann Arbor, MI). Dosimetry was performed using an ionization chamber connected to an electrometer system that is directly traceable to a National Institute of Standards and Technology calibration.

### Clonogenic survival assay

For the clonogenic assay, cells were plated into 6-well cell culture plates at a concentration previously determined by plating efficiency. After overnight incubation, cells were exposed to a range of IR doses with or without pretreatment, as indicated in individual experiments. The cells were allowed to grow for an additional 7 days to form colonies and then stained with 0.1% crystal violet. Colonies with more than 50 cells were counted as surviving colonies and normalized with the colony number observed in nonirradiated cells.

### Immunofluorescence

Cells were placed on glass coverslips in 6-well plates. After the indicated treatment, cells were fixed with absolute methanol at −20°C for 5 min. Cells were blocked in 0.5% (v/v) Triton X-100 in PBS and 3% (w/v) bovine serum albumin (BSA) and then incubated with anti-Ki67 (MIB-1) (Dako, Glostrup, Denmark) or anti-p65 (ser15, Cell Signaling Technology). Cells were then washed three times, incubated with FITC-conjugated secondary antibody and stained with Hoechst 33342 for visualization of DNA content and mitotic figures. Images were taken using a QImaging ExiAqua monochrome digital camera attached to a Nikon Eclipse 80i Microscope (Nikon, Melville, NY, USA) and visualized with QCapturePro software.

### Immunoblotting

Cells were harvested in RIPA buffer and briefly sonicated. Protein lysates were separated by 10% to 15% SDS–PAGE and transferred to a polyvinyl difluoride membrane (Immobilon) (Millipore, Billerica, MA, USA). Membranes were blocked in 0.1 M Tris (pH 7.5), 0.9% NaCl and 0.05% Tween-20 (TBS-T) with 5% nonfat dry milk. Membranes were incubated with anti-phospho-NFκB p65 (Ser536) or (ser15) (Cell Signaling), anti-phospho-IκB-α (Ser32) (Cell Signaling), anti-IKK-α (p45) (Millipore), anti-IKK-β (Millipore), anti-p16 (BD Biosciences), anti-p21 (BD Biosciences), anti-p53 (Ser15) (Cell Signaling), and anti-p53 (Cell Signaling, clone 7F5). GAPDH (Millipore) served as a loading control. The reaction was visualized using ECL SuperSignal West Pico Substrate (Pierce Biotechnology, Waltham, MA, USA).

### Flow cytometry

Cell cycle distribution was accessed by propidium iodide staining. After treatment with Emetine, cells were harvested and fixed with 70% ethanol on ice for 2 hours. The cell pellet was resuspended in 0.5 mL PBS containing 0.25% Triton X-100 for permeabilization and incubated for 15 minutes on ice. Cells were then incubated with PBS containing propidium iodide (Sigma-Aldrich; 20 μg/mL) and RNase solution (Sigma-Aldrich; 10 μg/mL) for 30 minutes at room temperature. The relative number of cells in different phases of the cell cycle were assessed by flow cytometry, and the percentages of cells in subG0/G1, G1, S and G2 were calculated.

MEC cancer stem cell-like cells were identified by aldehyde dehydrogenase (ALDH) activity using flow cytometry. The Aldefluor kit (StemCell Technologies, Durham, NC, USA) was used according to the manufacturer's instructions to identify cells with high ALDH enzymatic activity. Cells with or without pretreatment, as indicated in individual experiments, were suspended with activated Aldefluor substrate (BODIPY amino acetate) or negative control (dimethylamino benzaldehyde, a specific ALDH inhibitor) for 45 minutes at 37^°^C. All samples were analyzed using a FACS Canto IV (BD Biosciences) at the University of Michigan Flow Cytometry Core.

### Statistical analysis

All statistical analysis was performed using GraphPad Prism (GraphPad Software, San Diego, CA). Statistical analysis of the mitosis assay, Ki67 staining, and flow cytometry were performed by one-way analysis of variance (ANOVA) followed by Tukey's multiple comparison tests. Asterisks denote statistical significance (*p < 0.05; **p < 0.01; ***p < 0.001; ****p < 0.0001; and NS *p* > 0.05).

## SUPPLEMENTARY MATERIALS FIGURE


