# Supplementary material for: Overcoming adaptive resistance in mucoepidermoid carcinoma through inhibition of the IKK-β/IκBα/NFκB axis
Source: Oncotarget. 2016 Sep 22;7(45):73032–44. doi: 10.18632/oncotarget.12195 (PMC5341961; doi:10.18632/oncotarget.12195)
Supplement: Supplementary file 1 [file oncotarget-07-73032-s001.pdf]

Overcoming adaptive resistance in mucoepidermoid carcinoma through inhibition of the IKK-β/IκBα/NFκB axis

Supplementary Materials

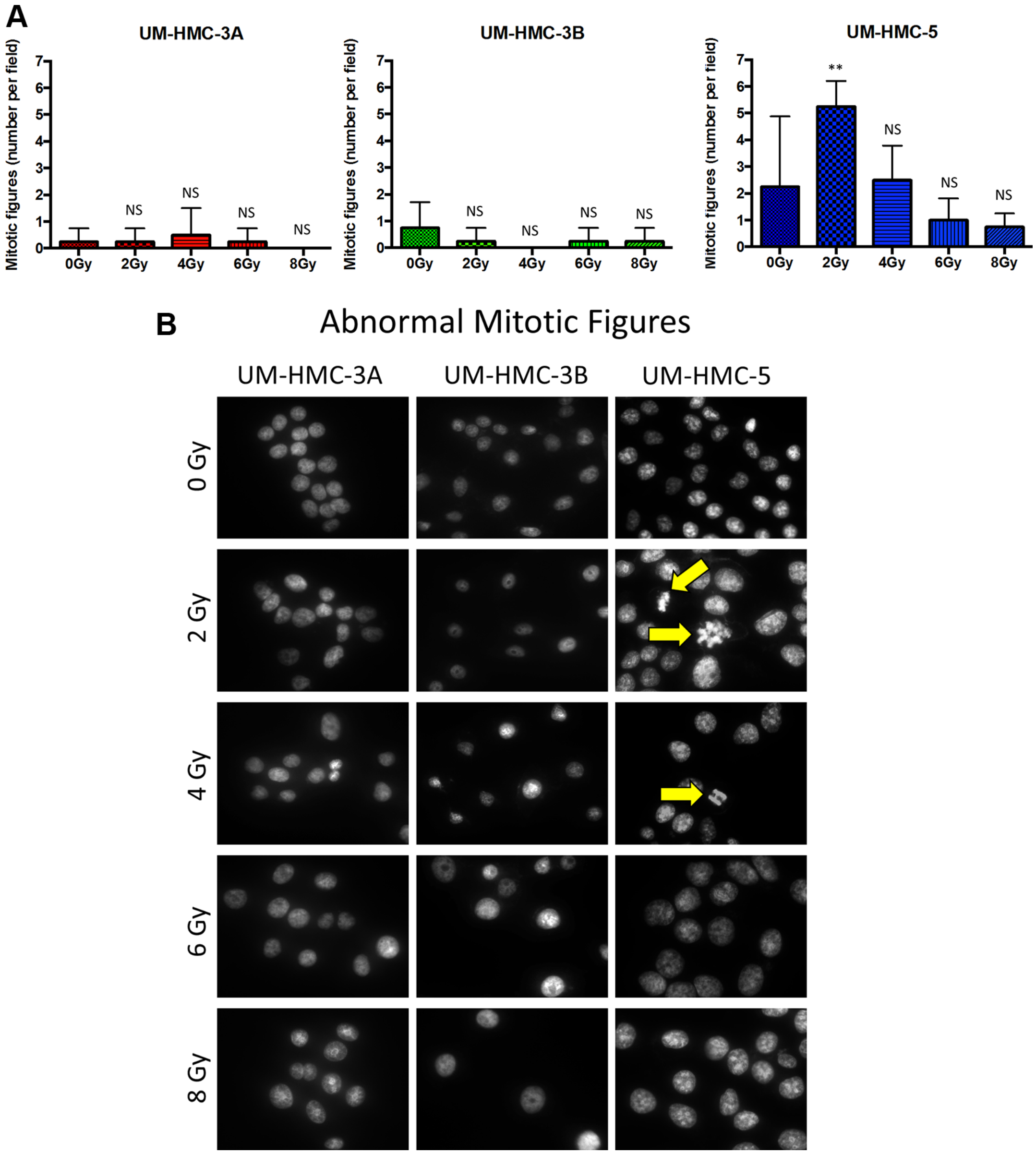

Supplementary Figure S1: IR stimulates abnormal mitotic figures in UM-HMC-5. There is a significant increase in mitotic figures in UM-HMC-5 upon exposure to 2 Gy of IR (*n* = 3, mean ± SD), as revealed by quantification of mitotic figures per field in all MEC cell lines. Representative images of MEC cell lines after IR (0–8 Gy) and staining with Hoechst 33342 for visualization of DNA content. Note the abnormal mitotic figures in UM-HMC-5 with 2 Gy and 4 Gy (yellow arrows).
